# Current Management and Treatment of Dry Eye Disease

**DOI:** 10.4274/tjo.69320

**Published:** 2018-12-27

**Authors:** Cem Şimşek, Murat Doğru, Takashi Kojima, Kazuo Tsubota

**Affiliations:** 1 Keio University Faculty of Medicine, Department of Ophthalmology, Tokyo, Japan

**Keywords:** Dry eye, treatment of dry eye, tear film breakup time

## Abstract

A better understanding of the pathophysiology and etiology of dry eye disease leads to more efficient management and treatment of the disease process. However, there is substantial variation among both clinicians and countries in terms of dry eye treatment modalities. The latest 2017 International Dry Eye Workshop II report aimed to reduce these differences and emphasized the use of a stepped care algorithm. The algorithm includes treatment forms ranging from artificial tear drops, the primary conventional treatment method, to the latest surgical applications. The aims of the algorithm are to restore homeostasis in the ocular surface, break the vicious cycle of inflammation, and ensure long-term ocular surface comfort.

## Introduction

Dry eye disease (DED) affects hundreds of millions of people worldwide. According to the 2017 International Dry Eye Workshop II (DEWS II) report, dry eye is “a multifactorial disease of the ocular surface characterized by a loss of homeostasis of the tear film, and accompanied by ocular symptoms, in which tear film instability and hyperosmolarity, ocular surface inflammation and damage, and neurosensory abnormalities play etiological roles.”^1^Typical clinically severe dry eye disease manifests with symptoms such as restriction of daily activities, pain, decreased wellness, and impairment in general health.^2,3,4^

Specifically, the term “multifactorial disease” states that DED is a complex functional disorder involving various findings and symptoms resulting from numerous complicated processes. The term “ocular surface” encompasses the tear film, lacrimal glands, meibomian glands, cornea, conjunctiva, and eyelids. Disruption of homeostasis refers to the tear film and ocular surface imbalances which accompany many symptoms in DED. Tear film instability, hyperosmolarity, inflammation, and damage, which are the main mechanisms contributing to the physiopathological process, were regarded as triggers of the vicious cycle occurring in DED.* Additionally, the *DEWS II final summary comprehensively emphasized that neuronal involvement and neurosensory abnormalities play an important role in the pathophysiology of DED.^5^

According to the pathophysiological classification, DED has two types, aqueous deficient dry eye (ADDE) and evaporative dry eye (EDE), and this classification is often used to make diagnosis and identify the treatment modality.^6^ This review compiles management and treatment options for dry eye disease. The DEWS II and the Asia Dry Eye Society (ADES) reports served as the basis of this review article.

A new treatment strategy developed in line with dry eye identification and diagnosis criteria is discussed in the DEWS II and ADES reports. It was emphasized in these newly published reports that more consideration should be given to etiological distinctions.^7^ Determining the necessity of the tear film for a healthy ocular surface and identifying tear film instability as a key factor in the diagnosis of dry eye brought attention to tear film layer stabilization, which led to the development of a new strategy called Tear Film-Oriented Therapy.^8^ This review emphasizes major innovations in dry eye treatment according to DEWS II and ADES reports. DED was previously believed to be largely due to tear deficiency, and accordingly, was treated by way of tear replacement, with artificial tears and punctum plugs.^9,10,11,12,13^ Recent advances in medical technology and our understanding of the pathophysiology, risk factors, and etiology of DED have contributed to an evolution in treatment strategies over time. Table 1 shows the treatment methods used in the management of DED in detail. In the years since publication of the original TFOS DEWS II Management and Therapy Report,^14^ there has been a growing realization of the important contribution of meibomian gland dysfunction (MGD) to both the symptoms and signs of DED.^15^

The management of DED is highly complicated because of its multifactorial etiology associated with many mechanisms.^16^ Therefore, when making a diagnosis of dry eye, clinicians should clearly determine the underlying etiology, such as EDE or ADDE, which are the mechanisms that cause DED, and/or other ocular surface diseases, and they should administer relevant treatments accordingly.^17,18^ In addition, neurotrophic keratopathy accompanied by neuropathic pain and symptoms should definitely be considered in differential diagnosis of patients with intense symptoms despite mild signs.^19^ Consequently, determining the main causes behind DED is critical for proper management.

The ultimate goal of DED treatment is to restore homeostasis of the ocular surface and tear film by breaking the vicious cycle of the disease.^20^ In addition to short-term therapies, it is also necessary to consider long-term treatment by taking into consideration the sequelae that can occur during the chronic disease process. The sequential treatment algorithm suggested in the DEWS II report should not be applied rigidly, but according to the benefit it will provide the patient. In the majority of DED patients, the general purpose should be to start treatment with the interventions most likely to be beneficial, and use more advanced and specific treatments that target the pathophysiology.

Management algorithms are structured to recommend a series of treatments according to disease stage, but the issue is complicated in DED because the disease often differs from patient to patient in both severity and nature.^21^ Treatment planning based on treatment algorithms is done according to disease severity. However, due to the coexistence of many factors in DED, strictly adhering to the algorithm system does not always work. For this reason, a higher stage of treatment can be applied in patients who do not respond to the treatment at the proposed stage and in patients with severe dry eye, or treatment recommended for the next stage can be added while continuing the previous stage of treatment. Approaches adopting dry eye treatment at home in the early stage can usually be performed with over-the-counter lubricants which are low-risk and easy for patients to obtain, but further advanced treatment options should be considered in advanced patients.^10,21,22,23^ In conclusion, the TFOS DEWS II management and therapy report presents a step-wise approach to the treatment of DED. Implementation of the management and therapeutic algorithm according to disease severity can be summarized in four steps.

The first step includes alteration of the local environment, patient education, dietary modifications (including oral essential fatty acid supplementation), identification and potential modification/elimination of offending systemic and topical medications, addition of ocular lubricants of various types (if MGD is present, then consider lipid-containing supplements), lid hygiene, and warm compresses.

If the treatments in the first step are insufficient, the second step is required. Treatments considered in the second step include tea tree oil treatment for Demodex, preservative-free artificial tears (to avoid the toxic effects of preservatives), punctal plugs, moisture chamber devices and goggles to maintain moisture and temperature, overnight ointment application, removing blockages from the meibomian glands using a warming and expression device (such as Lipiflow), intense pulsed light therapy for MGD, and topical administration of drugs such as corticosteroids, antibiotics, secretagogues, non-glucocorticoid immunomodulators (cyclosporine and tacrolimus^24^), LFA-1 antagonist drugs (lifitegrast), and oral macrolide or tetracycline antibiotics.

If the above treatment options are inadequate, oral secretagogues, autologous/allogenic serum eye drops, rigid and soft contact lenses need to be considered in addition as a third-step treatment.

If there is clinical evidence of more severe complications associated with the dry eye presentation, the clinician will need to consider additional treatments in the fourth step, such as application of topical corticosteroid for longer duration, amniotic membrane grafts, surgical punctal occlusion, and other surgical approaches (e.g., tarsorrhaphy, salivary gland transplantation).^7^

**Figure 1:**
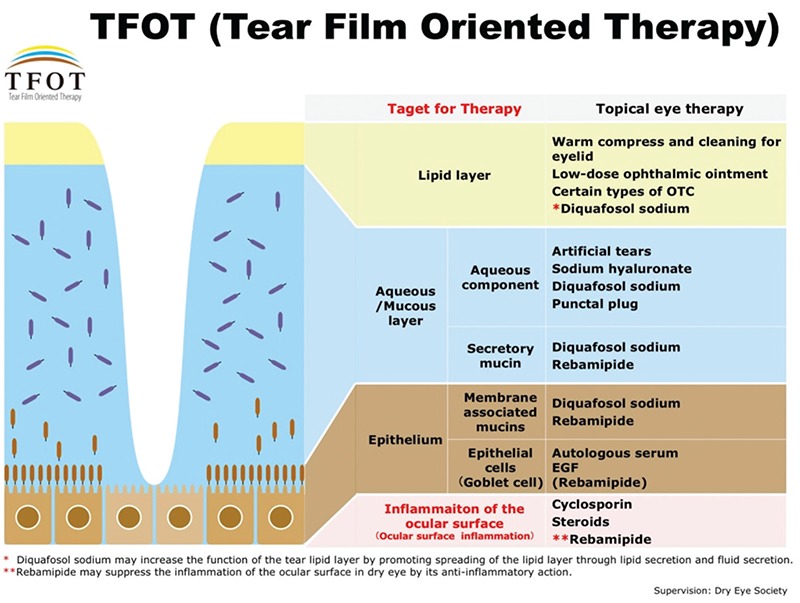
A newly developed treatment strategy for dry eye disease: “tear film-oriented therapy” (used with permission from the Dry Eye Society Japan)

In summary, dry eye diagnosis and treatment are evolving. The basic mechanism leading to the disease is still not known exactly. Accordingly, a global consensus has not been established in the diagnosis and treatment of the disease. Etiology-oriented treatment has gained importance in the meetings held by ADES and TFOS, and ADES has acknowledged the “Tear Film Layers-Oriented Therapy” protocol. The ADES consensus recommends that the deficient layer of the tear film should be replaced accordingly and the underlying problem should be addressed directly (Figure 1). Since it is very difficult to classify dry eye treatment within strict rules and base it only on evidence-based studies, each patient should be evaluated individually and patient-specific treatment plans should be made.

## Figures and Tables

**Table 1:**
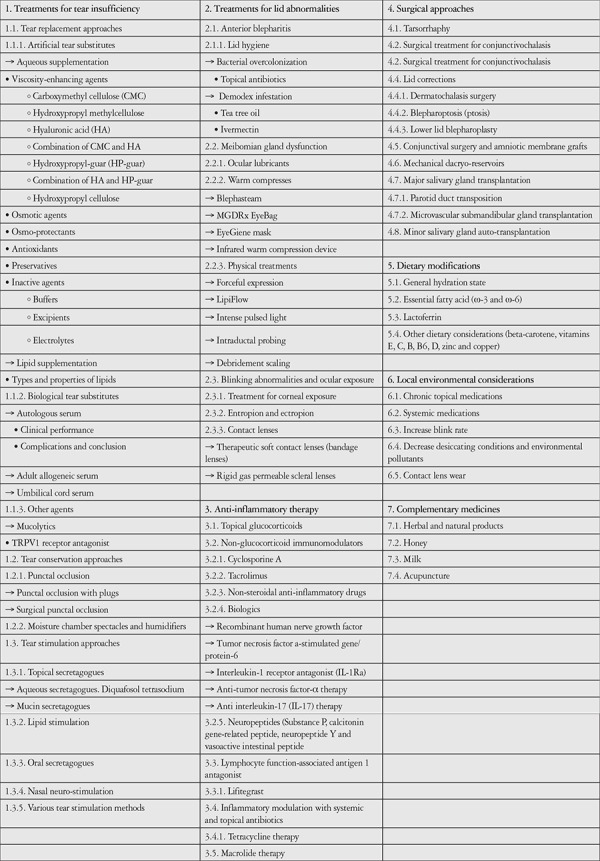
Detailed proposed treatment methods of dry eye disease
